# Deep learning-based reconstruction improves MRI image quality and diagnostic performance for carotid atherosclerotic plaques

**DOI:** 10.3389/fneur.2026.1793953

**Published:** 2026-04-17

**Authors:** Xixiang Chen, Changsheng Liu, Xuefang Lu, Weiyin Vivian Liu, Yabing Huang, Yunfei Zha, Zuneng Lu

**Affiliations:** 1 Department of Radiology, Renmin Hospital of Wuhan University, Wuhan, China; 2 China MR Research, GE Healthcare, Beijing, China; 3 Department of Pathology, Renmin Hospital of Wuhan University, Wuhan, China; 4 Department of Neurology, Renmin Hospital of Wuhan University, Wuhan, China

**Keywords:** carotid atherosclerosis, deep learning reconstruction, diagnostic accuracy, image quality, magnetic resonance imaging

## Abstract

**Objective:**

To evaluate the image quality and diagnostic performance of fast magnetic resonance imaging with deep learning-based reconstruction (MRI_DLR_) for carotid atherosclerotic plaques. We compared MRI_DLR_ to conventional MRI (MRI_C_) and fast MRI without deep learning-based reconstruction (MRI_Fast_).

**Methods:**

Sixty-nine patients were recruited between May 2022 and April 2024. Imaging was performed using a 3.0 T MRI system (SIGNA™ Architect, GE Healthcare) with a 19-channel head–neck coil. Image quality was assessed objectively [measuring signal-to-noise ratio (SNR) and contrast-to-noise ratio (CNR)], subjectively (evaluating overall image quality, noise, contrast, artifacts, sharpness and diagnostic performance). All statistical analyses were performed using R software version 4.4.2, with agreement among results assessed using Cohen’s *κ*.

**Results:**

The average scanning time was reduced by 12.4 min (approximately 63.2%) for MRI_DLR_ and MRI_Fast_ compared to MRI_C_. The SNR of T_1_ and T_2_ sequences for MRI_DLR_ and MRI_C_ showed significantly higher than MRI_Fast_ (*p* < 0.05), and the SNR of T_1_ and T_2_ sequences for MRI_DLR_ was significantly higher than MRI_C_. The SNR of PD sequences for MRI_DLR_ and MRI_C_ showed significantly higher than MRI_Fast_ (*p* < 0.05), and there was not significantly different between MRI_DLR_ and MRI_C_ (*p* > 0.05). The CNR of T_1_, T_2,_ and PD sequences for MRI_DLR_ and MRI_C_ showed significantly higher than MRI_Fast_ (*p* < 0.05), and there was not significantly different between MRI_DLR_ and MRI_C_ (*p* > 0.05). Subjective image quality was significantly higher for MRI_DLR_ than MRI_C_ and MRI_Fast_ (*p* < 0.05), and MRI_C_ was higher than MRI_Fast_ (*p* < 0.05). Comparing with MRI_C_ and MRI_Fast_, MRI_DLR_ was more effective in diagnosing hemorrhage, lipid nuclei, calcification, fiber cap disruption, and vulnerable plaques, and showed greater concordance with surgical pathology findings.

**Conclusion:**

MRI_DLR_ can offer superior image quality and diagnostic performance for carotid atherosclerotic plaques while reducing scanning time. So it enhances diagnostic accuracy and lessens the examination burden on patients which will contribute to the diagnosis and treatment of stroke.

## 1 Introduction

Ischemic stroke is globally recognized as a leading cause of mortality and disability, with carotid atherosclerosis (CAS) as a key risk factor (1). The pathophysiological basis of CAS involves lipid deposition, collagen fibers, calcium salts, and other substances in the intimal layer of the carotid artery, leading to atheromatous plaques that can cause vascular stenosis or occlusion, ultimately impairing cerebral blood flow (2). Epidemiological evidence links approximately 23% of ischemic strokes to carotid artery stenosis (3). The prevalence of carotid atherosclerosis rises with age, particularly in middle-aged, elderly individuals, and those with comorbidities such as hypertension and diabetes (4, 5). Early detection and thorough evaluation of carotid atherosclerosis plagues are crucial for stroke prevention.

With the development of advanced imaging technologies, the capacity of non-invasive medical imaging modalities to observe and quantify CAS has significantly improved (6, 7). MRI has become an important tool for assessing CAS plaques (8). Because of its high resolution and sensitivity to soft tissues, MRI provides detailed information about carotid plaques’ three-dimensional morphology, structure, and composition, making it invaluable for identifying vulnerable plaques and predicting stroke risk (9). However, current MRI protocols still have limitations. While conventional MRI produces relatively clear images, it requires a longer acquisition time (about 35 min), leading to higher time costs (10). On the other hand, fast MRI can generate images more quickly, but it is limited by a suboptimal SNR and CNR and poor image quality (11). Thus, shortening scanning time while improving MRI image quality has become a focus of attention and research for clinical diagnosticians.

Conventional methods to reduce acquisition time include parallel imaging and compressed sensing techniques (12, 13). Parallel imaging reduces the acquisition time by increasing acceleration factors, but with the increasing acceleration factor, the SNR decreases significantly (12). CS can accelerate scan because of redundancy of imaging data; however, it has the disadvantages of longer processing times and unnatural appearance of images (13). Recently, deep learning algorithms have significantly advanced in the field of medical imaging, particularly high-resolution MRI reconstruction techniques (14). Deep learning-based reconstruction (DLR) algorithms, typically employing convolutional neural network architectures such as U-Net, have been developed to denoise images and enhance spatial resolution by learning complex relationships between undersampled and fully-sampled data. These techniques have demonstrated significant improvements in SNR, CNR, and overall image quality in multiple anatomical regions, including brain imaging for gliomas (15) and cardiac imaging for late gadolinium enhancement (16).

Given the demonstrated efficacy of DLR in other anatomical regions, its application to carotid atherosclerotic plaque imaging holds promise for addressing the inherent trade-off between scan time and image quality (17–19). Deep learning-based approaches have shown potential in carotid artery segmentation and plaque characterization, suggesting that DLR could improve the visualization of plaque components critical for stroke risk assessment (20).

This study aims to comprehensively evaluate the image quality and diagnostic performance of fast magnetic resonance imaging with deep learning-based reconstruction (MRI_DLR_) in carotid atherosclerotic plaques compared to conventional MRI (MRI_C_) and fast MRI (MRI_Fast_), using a multi-dimensional framework that combines objective metrics (SNR, CNR) and subjective reader scores (including overall image quality, noise, contrast, artifacts, and sharpness). Key diagnostic parameters assessed include the identification of intraplaque hemorrhage (IPH), lipid-rich necrotic core (LRNC), calcification (CA), fibrous cap rupture (FCR), and vulnerable plaques. The study is expected to provide evidence supporting new clinical diagnostic techniques for carotid atherosclerosis plaques and contribute to the prevention and treatment of stroke.

## 2 Methods

### 2.1 Participants

Sample size was estimated *a priori* based on the primary outcome of qualitative reader scores. Using PASS 2021 software, assuming a mean score of 4.5 in the MRI_DLR_ group and 3.5 in the MRIFast group, with a common standard deviation (*σ*) of 1.2, a two-sided *α* of 0.05, and a power (1-*β*) of 0.80, a minimum of 52 patients was required. To account for potential dropouts or failed scans, a total of 69 patients who visited the Department of Neurology or Neurosurgery at Renmin Hospital of Wuhan University between May 2022 and April 2024 were recruited, including 58 males and 11 females. Inclusion criteria were: (1) carotid plaque detected by carotid vascular ultrasound or computed tomography angiography (CTA) with the plaque thickness greater than 3 mm; (2) age between 18 and 80 years; (3) ability to cooperate with MRI examination and provide signed informed consent. Exclusion criteria were: (1) contraindications to MRI scanning; (2) severe heart, liver, or kidney diseases; (3) poor image quality or artifacts hindering diagnosis; (4) carotid artery vascular abnormalities not related to atherosclerosis; (5) incomplete scan sequences. After excluding four patients due to poor image quality (primarily caused by motion artifacts), 65 patients (54 men and 11 women; mean age = 63 ± 11.4 years) were ultimately included.

All study procedures were conducted following the ethical standards as laid down in the 1964 Declaration of Helsinki and its later amendments, and informed consent was obtained from all participants.

### 2.2 MRI protocol

Imaging was performed using a 3.0 T MRI system (SIGNA™ Architect, GE Healthcare) with a 19-channel head–neck coil. Patients were positioned supine with their head and neck secured to minimize movement during the scan. Patients were instructed to maintain calm, steady breathing, and avoid swallowing to ensure image clarity and reduce motion artifacts. The multi-contrast MRI protocol included the following sequences: a bright blood 3D time-of-flight (3D TOF) magnetic resonance angiography and 2D dark blood sequences comprising T_1_-weighted imaging (T_1_WI), T_2_-weighted imaging (T_2_WI), and Proton Density Weighted Imaging (PDWI). The specific scan parameters are presented in Table 1. A contrast agent (gadolinium diamine injection) was administered to 48 patients (who are not allergic to gadolinium-based substances and do not have severe renal failure), at a dose of 0.2 mmoL per kilogram of body weight, followed by 20 mL flush of normal saline at an infusion rate of 2.5 mL/s. Delayed T_1_WI was performed approximately 5 min post-injection to capture contrast enhancement within the vascular system. Fat suppression was applied to all sequences, and all MR imaging was centered on the carotid artery bifurcation with a longitudinal coverage of 24 mm.

**Table 1:** MRI scan sequence parameters.

Parameters	3D-TOF	Conventional sequences			Fast sequences		
		2D-T_1_WI	2D-T_2_WI	2D-PD	2D-T_1_WI	2D-T_2_WI	2D-PD
TR (ms)	21	600	2,750	2,250	600	2,750	2,250
TE (ms)	3.4	20	85	25	20	85	25
FOV (cm)	16 × 16	14 × 14	14 × 14	14 × 14	14 × 14	14 × 14	14 × 14
Thickness/gap (mm)	1/0	2/0	2/0	2/0	2/0	2/0	2/0
Acquisition matrix	320 × 256	256 × 256	256 × 256	256 × 256	256 × 256	256 × 256	256 × 256
Echo-train length	1	4	10	8	4	10	8
Bandwidth	20.83	31.25	31.25	31.25	31.25	31.25	31.25
Number of excitations	2	6	6	6	2	2	2
Acquisition time	5.30	7.12	6.42	5.30	2.34	2.29	1.54

Compared with conventional sequences, fast sequences achieve scanning acceleration primarily through reducing the number of excitation pulses (NEX) from 6 to 2, the other parameters are same DLR technology is then used to compensate for the reduction in signal-to-noise ratio and image artefacts caused by accelerated acquisition.

A commercially available DLR algorithm (AIR™ Recon DL, GE Healthcare) (21) was applied to all 2D fast sequences. This algorithm employed a deep convolutional neural network based on a U-Net architecture, trained using a supervised learning approach with over 10,000 clinically acquired MRI datasets. The network maps input images with low SNRand artifacts to high-SNR, artifact-free reference images acquired with longer scan times. The algorithm identifies up to 4.4 million features from the acquired image data directly on the MR console immediately after scanning, thereby reducing both noise and Gibbs artifacts. It is applicable across multiple anatomical regions and compatible with various pulse sequences, contrast weightings, field strengths, and coil configurations (22, 23). The software integrates adjustable denoising levels (low, medium, high). In this study, the medium denoising level was routinely applied, as it generates images most closely resembling standard acquisitions. Potential algorithm-specific artifacts include mild oversmoothing in regions with extremely low SNR and, in rare cases, a “plastic-like” appearance at high-contrast boundaries; however, neither was observed in our carotid artery applications. Further technical details and validation have been described in our previous work (23–25).

### 2.3 Image analysis

All images of the 65 participants in DICOM format were securely transferred to GE advanced workstation for anonymization of participant identifiers to ensure confidentiality. Images from different sequences (conventional, fast, and DLR reconstructions) for the same patient were separated and presented randomly during different reading sessions. Two experienced radiologists (6 and 15 years of expertise in imaging diagnosis, respectively) independently reviewed the images, blinded to all clinical information. Both readers were unaware of the reconstruction type (MRI_Fast_, MRI_DLR_, or MRI_C_) during regions of interest (ROI) placement and subjective scoring.

#### 2.3.1 Objective evaluation of image quality

To quantify SNR and CNR, three circular regions of interest (ROI) with a minimum area of 1 mm^2^ were placed on the plaque, ipsilateral sternocleidomastoid muscle, and background at the same plane by two readers, avoiding regions with obvious artifacts and cavities. When patients had multiple plaques, the plaque with the largest cross-sectional area was selected for measurement to ensure consistency. The measurements were repeated three times, and the average was reported. The inter-reader agreement for ROI measurements was assessed using intraclass correlation coefficients (ICC > 0.85 for all measurements). SNR and CNR were calculated using the following equations:

SNR = SI_plaque_ /SD_background_

CNR = | SI_plaque_ − SI_muscle_ |/ SD_background_

Where SI_plaque_ represents mean signal intensity of plaque, while SD_background_ represents signal intensity standard deviation of background, SI_muscle_ represents mean signal intensity of ipsilateral sternocleidomastoid muscle.

#### 2.3.2 Subjective image quality

Prior to the formal scoring, the two readers underwent a training session to familiarize themselves with the image characteristics of DLR-reconstructed images compared with conventional images. A pilot study was conducted using images from 10 randomly selected patients (covering all three reconstruction methods: MRI_C_, MRI_Fas_t, and MRI_DLR_). The two radiologists jointly reviewed these images, discussed the specific features associated with each grade (1–5) of the scoring criteria (e.g., noise level, image sharpness, and severity of artifacts), with particular emphasis on recognizing potential DLR-specific image features such as noise reduction patterns and texture alterations. Following this training, they reached a consensus on the interpretation of the scoring criteria, which served as the reference standard for the formal scoring. This process ensured consistent scoring between the two readers despite their different levels of experience.

Two readers, respectively, estimated the image quality of all 2D sequences using a five-point Likert scale (1 = unacceptable, hindering diagnosis; 2 = poor; 3 = moderate; 4 = good; 5 = excellent). Images rated as poor (score <2) by either reader were excluded from the final analysis. The image quality evaluation indicators included contrast, noise, artifacts, sharpness, and overall quality. The scoring results were analyzed statistically.

#### 2.3.3 Evaluation of diagnostic effectiveness

The two readers identified IPH, LRNC, CA, and FCR based on signal intensity across all sequences, using previously published criteria for assessment (26). The diagnostic criteria were as follows: For fresh IPH (<1 week), a high signal was observed in 3D-TOF and T_1_WI sequences, while the signal in T_2_WI and PDWI sequences was equal to or low. Recent IPH (1–6 weeks old) showed a high signal in all sequences (27–29). LRNC displayed an equal signal in 3D-TOF, a low signal in T_2_WI, and a high or equal signal in T_1_WI and PDWI (30). CA exhibited a low signal across all sequences. A complete thick fibrous cap appeared as a uniform, continuous low signal near the vascular lumen in 3D-TOF sequence,but a higher signal in T_2_WI, T_1_WI, and PDWI sequences (31). FCR appeared as an interrupted low signal in 3D-TOF sequence, with an irregular lumen observed in all sequences (32). In cases of disagreement between the two readers regarding plaque component identification, a consensus was reached through discussion. Plaque vulnerability was evaluated using the American Heart Association (AHA) plaque typing methodology based on modified classification standards for MRI (26). The typesI-II, III, VII, and VII are categorized as stable plaque, and the types IV-V and VI are classified as vulnerable plaque.

Twenty-two patients underwent carotid endarterectomy (17 men and 5 women; mean age = 67 ± 13 years). The median time interval between MRI examination and surgery was 7 days with the interquartile range (*IQR* 4–11 days). The pathological results were analyzed by an experienced pathologist with 15 years of expertise in pathological diagnosis, who was blinded to the MRI findings.

### 2.4 Statistical analysis

All statistical analyses were performed using R software version 4.4.2, *p*-value < 0.05 was considered statistically significant. Nomality was assessed using the Shapiro–Wilk test, supplemented by skewness and kurtosis statistics. These tests indicated non-normal distribution for all continuous variables. Consequently, continuous variables were reported as medians with interquartile range (*IQR*, 25th–75th percentiles). Qualitative image analysis results were presented as mean and median with *IQR*. The Kruskal–Wallis *H*-test was used to evaluate group differences, with *post-hoc* comparisons performed using the Nemenyi procedure, a non-parametric multiple comparison test that controls the family-wise error rate by comparing rank sums of all possible pairs based on the critical difference derived from the Studentized range distribution. Inter-reader agreement between the two readers and agreement between readers and surgical findings were assessed using Cohen’s *κ*, with 95% confidence intervals (33). The interpretation was as follows: *κ* < 0 indicated observational consistency is less than opportunity consistency; 0.20 or less, poor agreement; 0.21–0.40, fair agreement; 0.41–0.60, moderate agreement; 0.61–0.80, substantial agreement; and greater than 0.80, almost perfect agreement.

## 3 Results

### 3.1 Patient characteristics

A total of 65 patients (54 men and 11 women; mean age = 63 ± 11.4 years) with complete MRI datasets were included, following the exclusion of four volunteers due to poor image quality. Clinical presentations comprised 15 asymptomatic patients (23.1%), 27 with transient ischemic attack (41.5%), and 23 with stroke (35.4%). Regarding the degree of carotid stenosis, 34 patients had mild to moderate stenosis (<70%) (52.3%) and 31 had severe stenosis (≥70%) (47.7%). Comorbidities included hypertension (*n* = 41), hyperlipidemia (*n* = 23), diabetes mellitus (*n* = 19), cigarette smoking (*n* = 23), and alcohol consumption (*n* = 19). Among them 22 patients who underwent carotid endarterectomy, they all were symptomatic and severe carotid artery stenosis (100%).whereas those in the non-surgical group were more likely to be asymptomatic or mild-to-moderate stenosis. More details can be found in Table 2.

**Table 2:** Characteristics of patients.

Variables	Non-surgical treatment (*n* = 43)	surgical treatment (*n* = 22)
Sex, male	37 (86%)	17 (77.3%)
Age, y	63 ± 11.4	67 ± 13.0
Hypertension	29 (67.4%)	16 (72.7%)
Hyperlipidemia	23 (53.5%)	13 (59.1%)
Diabetes	19 (44.2%)	9 (40.9%)
Smoking	23 (53.5%)	14 (63.6%)
Drinking	12 (27.9%)	13 (59.1%)
No symptom	15 (34.9%)	0 (0.0%)
TIA	17 (39.5%)	10 (45.5%)
Stroke	11 (25.6%)	12 (54.5%)
Mild to moderate carotid artery stenosis	34 (79.1%)	0 (0.0%)
Severe carotid artery stenosis	9 (20.9%)	22 (100.0%)

### 3.2 Scanning time

Table 1 shows the time required for conventional sequences and fast sequences. This study’s average scan time for T_1_, T_2_, and PD sequences with conventional MRI was 19.4 min. In contrast, fast MRI or DLR MRI required only approximately 7 min, representing an average reduction of 12.4 min, saving 63.2% of scan time per patient. The fast sequences are significantly efficient than conventional sequences, which will greatly enhance the patient experience and improve diagnostic efficiency in hospitals.

### 3.3 Objective evaluation of image quality

**Figure 1:**
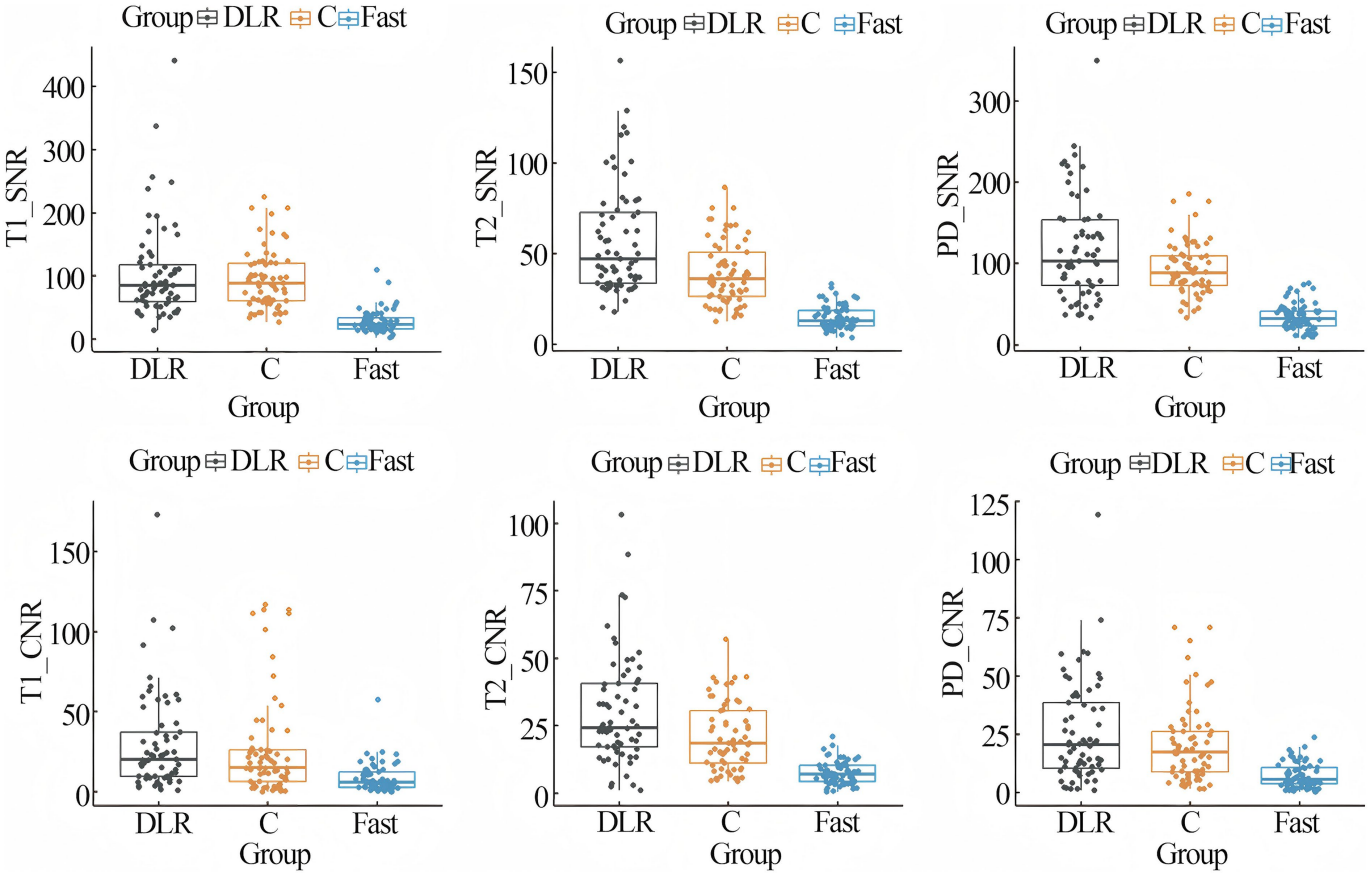
Box plots and scatter plots of CNR and SNR. The box distinctly outlines the interquartile range of the data, while the horizontal line within the box accurately indicates the median value. Each point in the scatter plot represented the precise measurement of an individual sample. DLR = fast MRI with deep learning reconstruction, C = conventional MRI, Fast = fast MRI without deep learning reconstruction.

The quantitative analysis of SNR and CNR for MRI_DLR_, MRI_C_ and MRI_Fast_ was summarized in Table 3. The SNR of T_1_ and T_2_ sequences for MRI_DLR_ and MRI_C_ showed significantly higher than MRI_Fast_ (*p* < 0.05), and the SNR of T_1_ and T_2_ sequences for MRI_DLR_ was significantly higher than MRI_C_. The SNR of PD sequences for MRI_DLR_ and MRI_C_ showed significantly higher than MRI_Fast_ (*p* < 0.05), and there was not significantly different between MRI_DLR_ and MRI_C_ (*p* > 0.05). The CNR of T_1_, T_2,_ and PD sequences for MRI_DLR_ and MRI_C_ showed significantly higher than MRI_Fast_ (*p* < 0.05), and there was not significantly different between MRI_DLR_ and MRI_C_ (*p* > 0.05). Box and scatter plots were constructed to visualize the SNR and CNR of MRI_DLR_, MRI_C_ and MRI_Fast_, as shown in Figure 1.

**Table 3:** Comparison of SNRs and CNRs for MRI_DLR_, MRI_C_, and MRI_Fast_.

Variables	MRI_DLR_, media [*IQR*]	MRI_C_, media [*IQR*]	MRI_Fast_, media [*IQR*]	*p*-value
SNR, dB				
T_1_	84.79 [59.28, 119.67]	89.22 [59.49, 120.99]	23.61 [16.36, 34.20]	<0.001^a^^,^^b^^,^^c^
T_2_	47.13 [33.69, 73.35]	36.13 [25.80, 51.71]	13.80 [10.28, 19.33]	<0.001^a^^,^^b^^,^^c^
PD	103.61 [72.70, 153.68]	88.49 [72.99, 110.17]	32.06 [23.01, 42.41]	<0.001^b^^,^^c^
CNR, dB				
T_1_	20.23 [9.68, 39.45]	15.11 [6.67, 26.62]	6.07 [2.77, 12.51]	<0.001^b^^,^^c^
T_2_	24.34 [16.73, 40.72]	18.57 [10.72, 30.66]	6.90 [4.22, 10.25]	<0.001^b^^,^^c^
PD	20.40 [10.01, 39.95]	17.35 [8.36, 27.16]	5.73 [3.40, 10.71]	<0.001^b^^,^^c^

MRI_DLR_ = fast MRI with deep learning reconstruction, MRI_C_ = conventional MRI, MRI_Fast_ = fast MRI without deep learning reconstruction.

a The Nemenyi *post-hoc* test found significant differences between MRI_DLR_ and MRI_C_.

b The Nemenyi *post-hoc* test found significant differences between MRI_DLR_ and MRI_Fast_.

c The Nemenyi *post-hoc* test found significant differences between MRI_C_ and MRI_Fast_.

### 3.4 Subjective evaluation of image quality

Table 4 presents the scoring data for MRI_DLR_, MRI_C_, and MRI_Fast_ evaluated by two readers. All scores(including overall image quality, noise, contrast, artifacts, and sharpness) of T_1,_ T_2,_ and PD sequences for MRI_DLR_ by two readers were significantly higher than MRI_C_ and MRI_Fast_ (*p* < 0.05), and MRI_C_ was significantly higher than MRI_Fast_ (*p* < 0.05).

**Table 4:** The image quality of MRI_DLR_, MRI_C_, and MRI_Fast_.

Sequence	Item	Reader 1				Reader 2			
		MRI_DLR_	MRI_C_	MRI_Fast_	*p*-value	MRI_DLR_	MRI_C_	MRI_Fast_	*p*-value
T_1_	Image quality	4.69 [5 (5,5)]	4.09 [4 (4,4)]	2.64 [3 (2,3)]	<0.001^a^^,^^b^^,^^c^	4.66 [5 (5,5)]	4.08 [4 (4,4)]	2.60 [3 (2,3)]	<0.001^a^^,^^b^^,^^c^
	Noise	4.71 [5 (5,5)]	4.07 [4 (4,4)]	2.36 [2 (2,3)]	<0.001^a^^,^^b^^,^^c^	4.62 [5 (5,5)]	4.09 [4 (4,4)]	2.35 [2 (2,3)]	<0.001^a^^,^^b^^,^^c^
	Contrast	4.63 [5 (5,5)]	4.12 [4 (4,4)]	2.86 [3 (3,3)]	<0.001^a^^,^^b^^,^^c^	4.60 [5 (5,5)]	4.12 [4 (4,5)]	2.80 [3 (3,3)]	<0.001^a^^,^^b^^,^^c^
	Artifacts	4.61 [5 (4,5)]	4.12 [4 (4,4)]	2.76 [3 (3,3)]	<0.001^a^^,^^b^^,^^c^	4.64 [5 (4,5)]	4.15 [4 (4,4)]	2.73 [3 (3,3)]	<0.001^a^^,^^b^^,^^c^
	Sharpness	4.77 [5 (5,5)]	4.07 [4 (4,4)]	2.87 [3 (3,3)]	<0.001^a^^,^^b^^,^^c^	4.74 [5 (5,5)]	4.04 [4 (4,4)]	2.81 [3 (3,3)]	<0.001^a^^,^^b^^,^^c^
T_2_	Image quality	4.51 [5 (4,5)]	4.12 [4 (4,4)]	2.53 [3 (2,3)]	<0.001^a^^,^^b^^,^^c^	4.53 [5 (4,5)]	4.07 [4 (4,4)]	2.55 [3 (2,3)]	<0.001^a^^,^^b^^,^^c^
	Noise	4.69 [5 (4,5)]	4.04 [4 (4,4)]	2.29 [2 (2,3)]	<0.001^a^^,^^b^^,^^c^	4.61 [5 (5,5)]	4.10 [4 (4,4)]	2.29 [2 (2,3)]	<0.001^a^^,^^b^^,^^c^
	Contrast	4.60 [5 (4,5)]	4.06 [4 (4,4)]	2.84 [3 (3,3)]	<0.001^a^^,^^b^^,^^c^	4.52 [5 (5,5)]	4.09 [4 (4,4)]	2.76 [3 (3,3)]	<0.001^a^^,^^b^^,^^c^
	Artifacts	4.35 [5 (4,5)]	4.22 [4 (4,5)]	2.83 [3 (2,3)]	<0.001^a^^,^^b^^,^^c^	4.38 [5 (4,5)]	4.13 [4 (4,5)]	2.75 [3 (3,3)]	<0.001^a^^,^^b^^,^^c^
	Sharpness	4.67 [5 (4,5)]	4.13 [4 (4,5)]	2.90 [3 (3,3)]	<0.001^a^^,^^b^^,^^c^	4.64 [5 (5,5)]	4.09 [4 (4,4)]	2.83 [3 (3,3)]	<0.001^a^^,^^b^^,^^c^
PD	Image quality	4.73 [5 (4,5)]	4.07 [4 (4,4)]	2.81 [3 (3,3)]	<0.001^a^^,^^b^^,^^c^	4.70 [5 (5,5)]	4.10 [4 (4,4)]	2.84 [3 (3,3)]	<0.001^a^^,^^b^^,^^c^
	Noise	4.67 [5 (4,5)]	4.06 [4 (4,4)]	2.69 [3 (2,3)]	<0.001^a^^,^^b^^,^^c^	4.63 [5 (5,5)]	4.05 [4 (4,4)]	2.67 [3 (2,3)]	<0.001^a^^,^^b^^,^^c^
	Contrast	4.76 [5 (4,5)]	4.04 [4 (4,4)]	2.87 [3 (3,3)]	<0.001^a^^,^^b^^,^^c^	4.75 [5 (5,5)]	4.06 [4 (4,4)]	2.81 [3 (3,3)]	<0.001^a^^,^^b^^,^^c^
	Artifacts	4.60 [5 (4,5)]	4.12 [4 (4,4)]	2.83 [3 (3,3)]	<0.001^a^^,^^b^^,^^c^	4.58 [5 (5,5)]	4.09 [4 (4,4)]	2.80 [3 (3,3)]	<0.001^a^^,^^b^^,^^c^
	Sharpness	4.78 [5 (4,5)]	4.09 [4 (4,4)]	2.86 [3 (3,3)]	<0.001^a^^,^^b^^,^^c^	4.73 [5 (5,5)]	4.12 [4 (4,4)]	2.81 [3 (3,3)]	<0.001^a^^,^^b^^,^^c^

The results are reported as mean [median (interquartile range)].

MRI_DLR_ = fast MRI with deep learning reconstruction, MRI_C_ = conventional MRI, MRI_Fast_ = fast MRI without deep learning reconstruction.

a The Nemenyi *post-hoc* test found significant differences between MRI_DLR_ and MRI_C_.

b The Nemenyi *post-hoc* test found significant differences between MRI_DLR_ and MRI_Fast_.

c The Nemenyi *post-hoc* test found significant differences between MRI_C_ and MRI_Fast_.

### 3.5 Evaluation of diagnostic effectiveness

**Figure 2:**
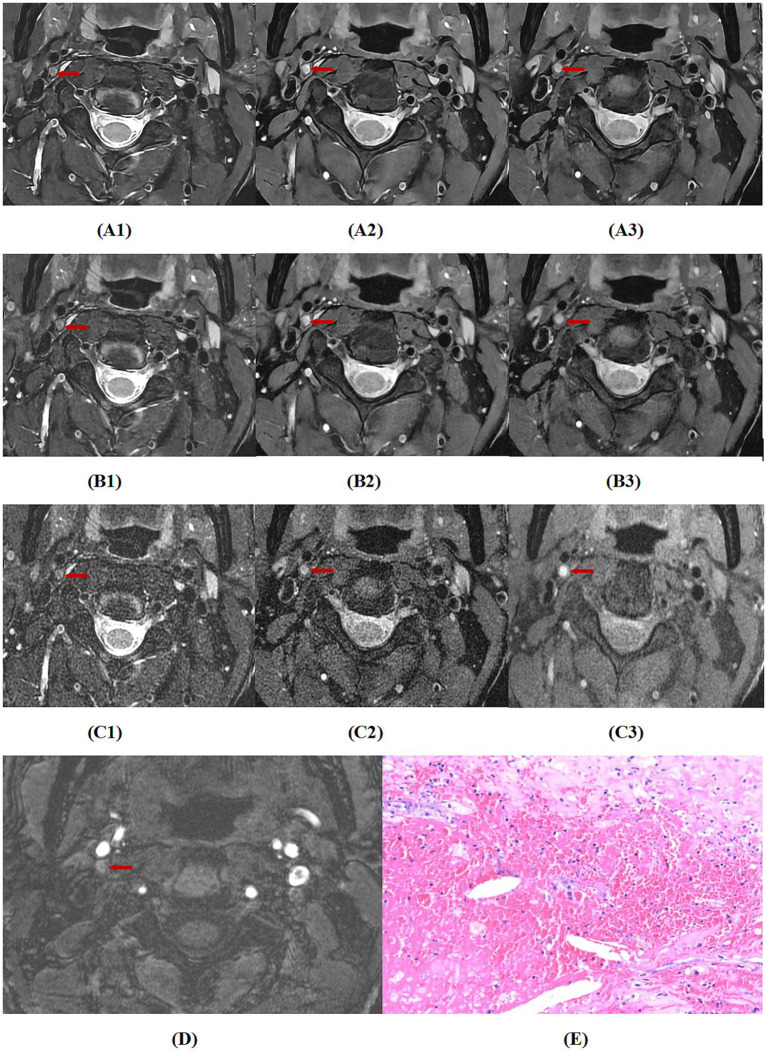
A case with a sudden onset of blurred vision in the right eye. **(A1–A3)** Respectively are MRI_DLR_ images of T_2_, PD, T_1_, **(B1–B3)**, respectively are MRI_C_ images of T_2_, PD, T_1_, **(C1–C3)**, respectively, are MRI_FAST_ images of T_2_, PD, T_1_, **(D)** is a 3D-TOF image, and **(E)** is a pathological section (100 × microscopy, HE staining). The MRI_DLR_ image quality is the best with the clearest plaque display; the MRI_C_ image quality is slightly worse; and the MRI_FAST_ image quality is the worst with a blurred plaque display. In the figure, the right internal carotid artery shows a round-like hemorrhagic plaque with high signals on 3D-TOF, T_2_, PD, and T_1_ (indicated by arrows), which was confirmed by the pathological findings.

**Figure 3:**
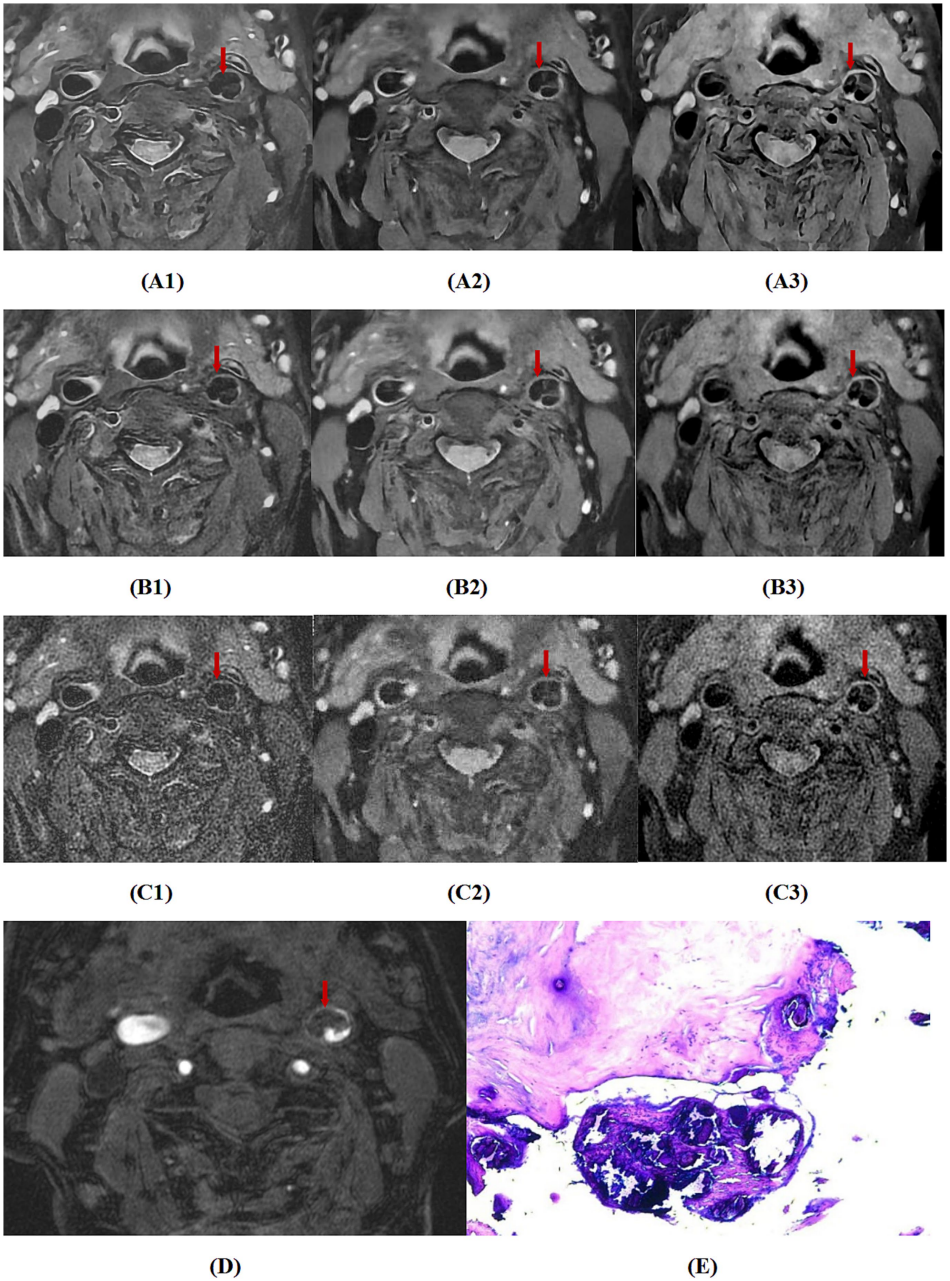
A case with sudden loss of vision in the left eye. **(A1–A3)** Respectively are MRI_DLR_ images of T_2_, PD, T_1_, **(B1–B3)** respectively are MRI_C_ images of T_2_, PD, T_1_, **(C1–C3)** respectively are MRI_FAST_ images of T_2_, PD, T_1_, **(D)** is a 3D-TOF image, and **(E)** is a pathological section (100 × microscopy, HE staining). The MRI_DLR_ image quality is the best with the clearest plaque display; the MRI_C_ image quality is slightly worse; and the MRI_FAST_ image quality is the worst with a blurred plaque display. In the figure, the left internal carotid artery shows a large calcified plaque with a low signal on 3D-TOF, T_2_, PD, and T_1_ (indicated by arrows), which was confirmed by the pathological findings.

**Figure 4:**
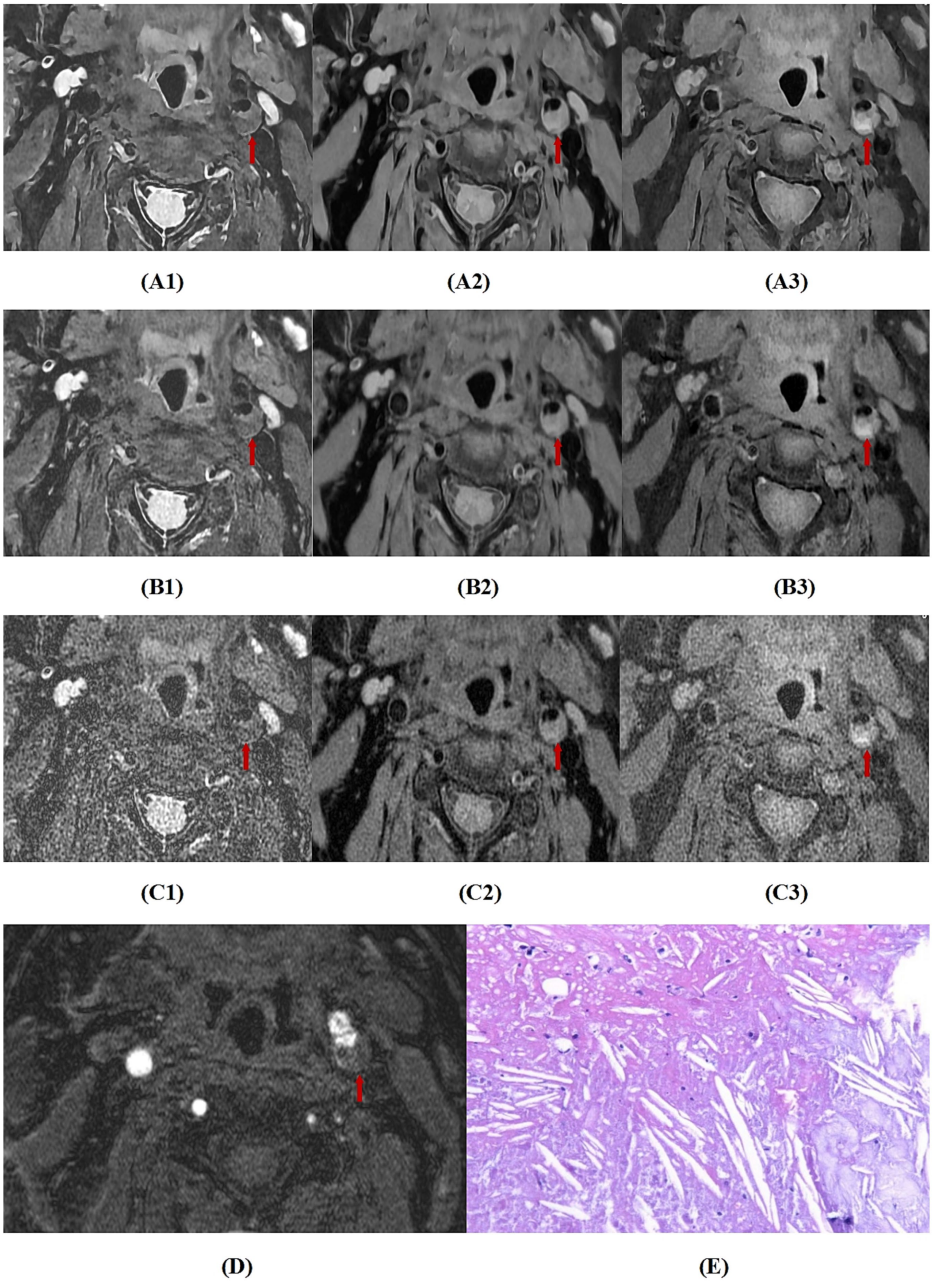
A case with sudden onset of right limb weakness. **(A1–A3)** respectively are MRI_DLR_ images of T_2_, PD, T_1_, **(B1–B3)**, respectively are MRI_C_ images of T_2_, PD, T_1_, **(C1–C3)**, respectively are MRI_FAST_ images of T_2_, PD, T_1_, **(D)** is a 3D-TOF image, and **(E)** is a pathological section (100 × microscopy, HE staining). The MRI_DLR_ image quality is the best with the clearest plaque display; the MRI_C_ image quality is slightly worse; and the MRI_FAST_ image quality is the worst with a blurred plaque display. In the figure, the left internal carotid artery shows a large lipid plaque with iso-signal on 3D-TOF, low signal on T_2_, and slightly high signal on PD and T_1_ (indicated by arrows), which was confirmed by pathological findings.

For the agreement in diagnosing bleeding, lipid nucleus, calcification, fibrous cap rupture, and vulnerable plaque between the two readers, the *κ* values of MRI_DLR_, MRI_C_, and MRI_Fast_ were greater than 0.80, almost perfect agreement. Among these, MRI_DLR_ showed the highest inter-reader agreement. Taking surgical pathology results as the gold standard, MRI_DLR_ demonstrate the highest diagnostic accuracy. For the diagnosis of bleeding, lipid nucleus, calcification and fibrous cap rupture, the agreement between reader l and pathological findings of MRI_DLR_ was virtually perfect (*κ* > 0.80); in the assessment of vulnerable plaques, the agreement between the two was high (*κ* = 0.782). In contrast, MRI_C_ and MRI_Fast_ showed lower agreement with pathological findings; in particular, MRI_Fast_ demonstrated only moderate agreement (*κ* = 0.532) in the identification of vulnerable plaques. The assessment results of the second reader were similar to those of the first. Detailed *κ* values are shown in Table 5. Figures 2–4, respectively, showed hemorrhagic plaque, calcified plaque,and lipid plaque, and they all revealed the MRI_DLR_ image quality is the best with the clearest plaque display.

**Table 5:** Agreement of MRI_C_, MRI_DLR,_ and MRI_Fast_ with surgeries and inter-reader.

Items	MRI_DLR_, Cohen’s *κ (95%CI)*	MRI_C_, Cohen’s *κ (95%CI)*	MRI_Fast_, Cohen’s *κ (95%CI)*
Reader 1 with surgeries			
Bleeding	0.904 (0.811–0.997)***	0.908 (0.819–0.977)***	0.723 (0.575–0.871)***
Lipid nucleus	0.891 (0.785–0.997)***	0.773 (0.624–0.922)***	0.645 (0.467–0.823)***
Calcification	0.927 (0.857–0.997)***	0.779 (0.631–0.927)***	0.704 (0.549–0.859)***
Fiber cap ruptured	0.904 (0.811–0.997)***	0.904 (0.811–0.997)***	0.814 (0.691–0.937)***
Vulnerable plaque	0.782 (0.630–0.934)***	0.636 (0.461–0.811)***	0.532 (0.369-0.695)***
Reader 2 with surgeries			
Bleeding	0.904 (0.811–0.997)***	0.904 (0.811–0.997)***	0.818 (0.701–0.935)***
Lipid nucleus	0.891 (0.785–0.997)***	0.773 (0.624–0.922)***	0.645 (0.467–0.823)***
Calcification	0.909 (0.821–0.997)***	0.817 (0.700–0.934)***	0.723 (0.575–0.871)***
Fiber cap ruptured	1.000 (1.000–1.000)***	0.904 (0.811–0.997)***	0.814 (0.691–0.937)***
Vulnerable plaque	1.000 (1.000–1.000)***	0.645 (0.467–0.823)***	0.463 (0.271–0.655)***
Inter-reader			
Bleeding	0.981 (0.962–1.000)***	0.962 (0.935–0.989)***	0.927 (0.891–0.963)***
Lipid nucleus	0.944 (0.904–0.984)***	0.914 (0.865–0.963)***	0.838 (0.768–0.908)***
Calcification	0.947 (0.917–0.977)***	0.929 (0.894–0.964)***	0.894 (0.852–0.936)***
Fiber cap ruptured	1.000 (1.000–1.000)***	0.959 (0.930–0.988)***	0.941 (0.907–0.975)***
Vulnerable plaque	0.903 (0.848–0.958)***	0.878 (0.819–0.937)***	0.818 (0.752–0.884)***

MRIDLR = fast MRI with deep learning reconstruction, MRI_C_ = conventional MRI, MRI_Fast_ = fast MRI without deep learning reconstruction.

*** = *p*<0.001. The agreement of pathological results with reader 1 and reader 2 diagnostic results were performed in 22 surgical patients. Inter-reader was performed in all patients.

## 4 Discussion

While DLR has shown promise in various anatomical regions, its application for comprehensive diagnostic evaluation of carotid atherosclerotic plaques—specifically regarding image quality improvement and diagnostic accuracy for key plaque components—has not been extensively studied. This study aims to address this gap by comprehensively evaluating the performance of MRI_DLR_ in carotid plaque assessment, demonstrating that MRI_DLR_ offers superior image quality and diagnostic performance with a shorter acquisition time.

MRI is a powerful diagnostic tool that reveals key characteristics of carotid plaques, including intraplaque hemorrhage, lipid-rich necrotic cores, calcification, fibrous caps, and inflammation (34). Accurately delineating these features is crucial for assessing plaque stability and stratifying the risk of future cerebrovascular events. High-resolution and high-sensitivity MRI techniques enable precise identification of plaque burdens. Before the advent of DLR, radiologists had two primary MRI options: Conventional MRI offered better image quality but required longer scanning times; and fast MRI provided quicker scanning but sacrificed image quality (35). Moreover, noise during MRI scanning can impact image quality and diagnostic accuracy. So improving image quality and reducing scanning time have been a major concern for radiologists.

The emergence of DLR technology provides radiologists with a new option, enabling higher-quality images to be obtained in a shorter time (36). The DLR technique is a deep convolutional neural network algorithm optimized through millions of fitting iterations. It is embedded at the raw data stage of MRI reconstruction, effectively reducing image noise while preserving detail and quality (37, 38). This offers advantages over both conventional MRI acquisitions that rely on an increased number of excitations (NEX) and filter-based denoising techniques such as Gaussian filtering (39, 40). Unlike filter-based methods, in which excessive denoising may lead to the loss of structural details, the proposed DLR technique preserves image fidelity while effectively suppressing noise (41). Furthermore, the DLR technique is directly and seamlessly compatible with existing MRI fast acquisition methods, effectively reducing the computational burden and accelerating the scanning process. This compatibility allows for the maintenance of high performance while achieving higher resolution and a more favorable SNR and CNR in the images. Consequently, MRI_DLR_ holds significant potential for broadening clinical applications.

In our study, MRI _DLR_ reduced scan time by approximately 63.2% compared to conventional MRI. The reduction of scan time is very important for patients and radiologists. Shorter examinations may enhance patient comfort to avoid involuntary movements—particularly important for elderly, children, and claustrophobic patients. And this is particularly crucial for our study, because carotid artery is more subject to the influence of breathing, swallowing, and vascular fluctuations. Moreover saving time has meaningful clinical implications beyond patient comfort. From an operational perspective, shorter scan times could increase patient throughput, reduce waiting lists, and improve scanner utilization efficiency—particularly relevant in high-volume centers where MRI access is often a bottleneck (42). While a formal cost-effectiveness analysis was beyond the scope of this study, the potential for reduced examination time and improved diagnostic confidence may translate into downstream healthcare savings by enabling more appropriate and timely treatment decisions.

Furthermore, the SNR and CNR of MRI_DLR_ were significantly higher than MRI_Fast_. As a deep learning reconstruction technique, MRI_DLR_ reduces image noise while preserving essential details, particularly in water imaging and background noise suppression, thereby improving the overall image quality (43). These findings further confirm the general advantages of DLR techniques in enhancing MRI image quality, consistent with conclusions observed in previous studies of the lumbar spine (44), shoulder joint (45), and prostate (46). Although the specific algorithmic implementations may vary across studies, these results collectively demonstrate that DLR technology can effectively improve image quality, supporting its potential for wider clinical application.

Our score results showed that the image scores for T_1_, T_2_, and PD sequences with DLR were consistently higher than those without DLR, similar to findings in a previous study on the knee (47). This suggests that DLR significantly enhances the native contrast and clarity of images, facilitating the initial identification and assessment of lesions’ location, morphology, and characteristics. To support this, we further analyzed the inter-reader diagnostic agreement for MRI_C_, MRI_DLR_, and MRI_Fast_ and the concordance between imaging diagnoses and surgical pathology outcomes. Consistent with our expectations, almost all MRI_DLR_ showed greater concordance in diagnosing critical features like hemorrhage, lipid-rich necrotic cores, calcification, fibrous cap disruption, and vulnerable plaque than MRI_C_ and MRI_Fast_. This indicates that the DLR technique possesses significant advantages in discerning the pathological characteristics of carotid plaques, which is crucial for informing clinical treatment decisions and assessing stroke risk. A comparative analysis of the actual images reveals that DLR images provide clearer visualization of plaque details, thereby enabling radiologists to more accurately evaluate plaque stability and potential risks.

These diagnostic improvements have direct implications for patient management. Accurate identification of vulnerable plaque features—particularly intraplaque hemorrhage and thin/ruptured fibrous caps—is critical for risk stratification and treatment planning. Patients with these high-risk features may benefit from more aggressive intervention, including timely endarterectomy or stenting, whereas those with stable plaque phenotypes could be managed conservatively with optimal medical therapy, avoiding unnecessary procedural risks. By improving the accuracy of plaque characterization, MRI_DLR_ could support more personalized treatment decisions, potentially reducing both overtreatment of stable disease and undertreatment of vulnerable plaques. Furthermore, clearer visualization of plaque morphology may aid surgeons in pre-procedural planning for endarterectomy, particularly in complex cases involving plaque extension or calcification.

Our study had several limitations. First, the small sample size (*n* = 69) and single-center design, combined with the lack of an external validation cohort, may limit the generalizability of our findings. In particular, only 22 patients (31.9%) underwent carotid endarterectomy with pathological correlation, introducing potential selection bias, as this surgical subgroup predominantly comprised symptomatic patients with severe stenosis. Second, we applied this technology only to a 3.0 T MRI system from a single vendor (GE Healthcare), and the DLR algorithm in this study is proprietary; results may not apply to other deep learning reconstruction methods; diagnostic efficacy on 1.5 T scanners and other manufacturer platforms should be evaluated in future studies. Third, we did not assess the impact of DLR on treatment decision-making or long-term clinical outcomes. Finally, further applications such as postoperative monitoring should be explored. These aspects will be addressed in follow-up studies to further validate the clinical effectiveness of MRI_DLR_ for detecting carotid atherosclerotic plaques.

## 5 Conclusion

In summary, DLR techniques in medical imaging are rapidly advancing, particularly in MRI image reconstruction (48, 49). Our results support the application of DLR technology in carotid plaques diagnosis, especially in enhancing image quality, improving diagnostic accuracy, and reducing scan time. These advancements are crucial for diagnosis and treatment of stoke. With the continuous advancement of deep learning algorithms and computational power, DLR techniques are expected to see broader clinical applications, such as cardiovascular diseases and oncology.

## Data Availability

The raw data supporting the conclusions of this article will be made available by the authors, without undue reservation.
