# Supplementary material for: Deep learning-based reconstruction improves MRI image quality and diagnostic performance for carotid atherosclerotic plaques
Source: Front Neurol. 2026 Apr 17;17:1793953. doi: 10.3389/fneur.2026.1793953 (PMC13132737; doi:10.3389/fneur.2026.1793953)
Supplement: Supplementary file 1 [file Table_1.docx]

**Supplementary files**

**Table S1. The Shapiro-Wilk test of SNRs and CNRs for MRI_DLR_, MRI_C_, and MRI_Fast_**

| **Items** | | **MRI_DLR_** |  | **MRI_C_** |  | **MRI_Fast_** |  |
| --- | --- | --- | --- | --- | --- | --- | --- |
|  |  | ***W*** | ***P*** | ***W*** |  | ***W*** | ***P*** |
| **SNR** |  |  |  |  |  |  |  |
|  | T1 | 0.781 | ＜0.001 | 0.928 | 0.001 | 0.817 | ＜0.001 |
|  | T2 | 0.884 | ＜0.001 | 0.946 | 0.007 | 0.935 | 0.002 |
|  | PD | 0.919 | ＜0.001 | 0.961 | 0.037 | 0.947 | ＜0.008 |
| **CNR** |  |  |  |  |  |  |  |
|  | T1 | 0.775 | ＜0.001 | 0.727 | ＜0.001 | 0.735 | ＜0.001 |
|  | T2 | 0.907 | ＜0.001 | 0.938 | 0.003 | 0.964 | 0.054 |
|  | PD | 0.867 | ＜0.001 | 0.863 | ＜0.001 | 0.922 | ＜0.001 |

**Table S2. The Shapiro-Wilk test for MRI_DLR_, MRI_C_, and MRI_Fast_ in readers’ score of image quality**

| **Sequence** | **Item** | **Reader1** | | | | | |  |  |  | **Reader2** | | | |
| --- | --- | --- | --- | --- | --- | --- | --- | --- | --- | --- | --- | --- | --- | --- |
|  |  | **MRI_DLR_** |  | **MRI_C_** |  | **MRI_Fast_** |  |  | **MRI_DLR_** |  | **MRI_C_** |  | **MRI_Fast_** |  |
|  |  | ***W*** | ***P*** | ***W*** | ***P*** | ***W*** | ***P*** |  | ***W*** | ***P*** | ***W*** | ***P*** | ***W*** | ***P*** |
| **T1** | Image quality | 0.622 | ＜0.001 | 0.215 | ＜0.001 | 0.617 | ＜0.001 |  | 0.432 | ＜0.001 | 0.453 | ＜0.001 | 0.257 | ＜0.001 |
|  | Noise | 0.611 | ＜0.001 | 0.257 | ＜0.001 | 0.597 | ＜0.001 |  | 0.472 | ＜0.001 | 0.409 | ＜0.001 | 0.409 | ＜0.001 |
|  | Contrast | 0.604 | ＜0.001 | 0.327 | ＜0.001 | 0.357 | ＜0.001 |  | 0.453 | ＜0.001 | 0.384 | ＜0.001 | 0.215 | ＜0.001 |
|  | Artifacts | 0.580 | ＜0.001 | 0.103 | ＜0.001 | 0.453 | ＜0.001 |  | 0.597 | ＜0.001 | 0.294 | ＜0.001 | 0.103 | ＜0.001 |
|  | Sharpness | 0.597 | ＜0.001 | 0.165 | ＜0.001 | 0.294 | ＜0.001 |  | 0.432 | ＜0.001 | 0.521 | ＜0.001 | 0.257 | ＜0.001 |
| **T2** | Image quality | 0.432 | ＜0.001 | 0.103 | ＜0.001 | 0.635 | ＜0.001 |  | 0.570 | ＜0.001 | 0.357 | ＜0.001 | 0.327 | ＜0.001 |
|  | Noise | 0.409 | ＜0.001 | 0.104 | ＜0.001 | 0.559 | ＜0.001 |  | 0.432 | ＜0.001 | 0.294 | ＜0.001 | 0.357 | ＜0.001 |
|  | Contrast | 0.432 | ＜0.001 | 0.103 | ＜0.001 | 0.409 | ＜0.001 |  | 0.453 | ＜0.001 | 0.260 | ＜0.001 | 0.257 | ＜0.001 |
|  | Artifacts | 0.384 | ＜0.001 | 0.104 | ＜0.001 | 0.548 | ＜0.001 |  | 0.621 | ＜0.001 | 0.165 | ＜0.001 | 0.165 | ＜0.001 |
|  | Sharpness | 0.549 | ＜0.001 | 0.113 | ＜0.001 | 0.622 | ＜0.001 |  | 0.432 | ＜0.001 | 0.327 | ＜0.001 | 0.165 | ＜0.001 |
| **PD** | Image quality | 0.635 | ＜0.001 | 0.357 | ＜0.001 | 0.409 | ＜0.001 |  | 0.357 | ＜0.001 | 0.506 | ＜0.001 | 0.165 | ＜0.001 |
|  | Noise | 0.616 | ＜0.001 | 0.327 | ＜0.001 | 0.616 | ＜0.001 |  | 0.384 | ＜0.001 | 0.432 | ＜0.001 | 0.357 | ＜0.001 |
|  | Contrast | 0.641 | ＜0.001 | 0.215 | ＜0.001 | 0.294 | ＜0.001 |  | 0.358 | ＜0.001 | 0.559 | ＜0.001 | 0.215 | ＜0.001 |
|  | Artifacts | 0.611 | ＜0.001 | 0.257 | ＜0.001 | 0.384 | ＜0.001 |  | 0.632 | ＜0.001 | 0.103 | ＜0.001 | 0.103 | ＜0.001 |
|  | Sharpness | 0.626 | ＜0.001 | 0.165 | ＜0.001 | 0.327 | ＜0.001 |  | 0.459 | ＜0.001 | 0.551 | ＜0.001 | 0.312 | ＜0.001 |
